# Genome-Scale Computational Biology and Bioinformatics in Australia

**DOI:** 10.1371/journal.pcbi.1000068

**Published:** 2008-08-29

**Authors:** Mark A. Ragan, Tim Littlejohn, Bruce Ross

**Affiliations:** 1 Australian Research Council Centre of Excellence in Bioinformatics; 2 Institute for Molecular Bioscience, The University of Queensland, Brisbane, Australia; 3 School of Information Technology and Electrical Engineering, The University of Queensland, Brisbane, Australia; 4 IBM Australia; 5 Microsoft Australia; University of California San Diego, United States of America

## Introduction

Australia enjoys a high international reputation for research in experimental genetics, molecular and cell biology, animal and plant sciences, biotechnology, medicine, biodiversity, and ecological modelling. Computational research is broadly established in these domain areas and others relevant to bioscience. Combined with strong traditions in mathematics and statistics, and national and state investment in computational infrastructure, computational biology Down Under is simply too rich and diverse to be done justice in these few pages. Here we (see Box 1 Authors' Biographies) focus more narrowly, attempting to provide a snapshot of bioinformatic and computational genome-scale biology in Australia circa 2008.

##

**Box 1. Authors' Biographies**

**Mark Ragan** is Professor at The University of Queensland, where he is Head of Genomics and Computational Biology at the Institute for Molecular Bioscience, and Adjunct Professor in the School of Information Technology and Electrical Engineering. He is also Director of the Australian Research Council (ARC) Centre of Excellence in Bioinformatics. Mark holds a BA in Biochemistry from the University of Chicago and a PhD in Biology from Dalhousie University. As a researcher with National Research Council Canada (1978–2000), he helped initiate and develop programs in bioactive natural products, molecular biology, genomics, and bioinformatics, including the *Sulfolobus solfataricus* genome-sequencing project and Canadian Bioinformatics Resource. Mark was appointed a Fellow of the Canadian Institute for Advanced Research (1993–2000, relinquished upon his move to Australia) and is a Fellow of the Linnean Society of London. He served as co-chair of ISMB-2003 and general chair of APBC-1, and will chair the upcoming GIW-2008.

**Tim Littlejohn** is a member of the IBM Healthcare Life Sciences team, and a member of the IBM Australia Diversity Council. After receiving his PhD in molecular genetics from the University of Melbourne, Tim served as director of informatics at the Organelle Genome Sequencing Project in Montréal, then returned to Australia to head the Australian national bioinformatics facility ANGIS. Tim formed the bioinformatics companies Entigen Inc. in 1998, and BioLateral Pty Ltd in 2001. He has been actively involved in the Australian bioinformatics and biotechnology industry for many years, supplying technologies and services to biotechnologists across Australia and around the world. Tim led the Australian Federal Government's Bioinformatics Industry Opportunity Taskforce (2001–2002) and was co-founder (and is currently President) of the national peak bioinformatics body Bioinformatics Australia.

**Bruce Ross** has worked in a variety of roles in biology and computing. He received a PhD in microbial genetics from the University of Melbourne, then spent ten years in public health research applying genetics technologies to the diagnosis and epidemiology of infectious diseases. Thereafter he moved to CSL Ltd, where he was involved in human vaccine research and development for eight years. During this time, Bruce led a genome sequencing program designed to identify vaccine and drug target candidates for a human bacterial pathogen. He supplemented his biological qualifications with a degree in Software Engineering from RMIT University, and became leader of the bioinformatics group at CSL. After this he moved to IBM, where he was responsible for solution architecture and implementation for life sciences industries for five years, leading the implementation of a clinical research data repository and a data integration platform based on the ENSEMBL codebase. In late 2007 Bruce accepted a position at Microsoft Health Solutions Group.

## Origins: Platforms and Policies

Areas within computational bioscience have been strongly established for several decades in Australia, i.e., the modelling of natural resource utilisation, ecosystem dynamics, and protein structures. Australia was not, however, a significant partner in the international genome mapping and sequencing consortia of the 1990s, and a review of bioinformatics in Australia published by one of us in 2000 [1] did not mention any research projects in genomic biology. The Australian Genome Research Facility (AGRF) was established in 1997 as part of the Federal Government's Major National Research Facilities (MNRF) program and offered DNA library preparation, automated fluorescent sequencing, and first-stage bioinformatic services. There was never a national genomics program, funding body, or educational or training initiative; the first two organismal genomes sequenced domestically, two strains of *Leptospira borgpetersenii* [2], were published only in 2006. Most Australian involvement in genomic sequencing has been, and remains, via collaboration with groups overseas, e.g., the United States Department of Energy's Joint Genome Institute, Baylor College of Medicine, and RIKEN Genomic Sciences Center.

Building on strength in underlying biological and biomedical research, Australia was more proactive in functional genomics, i.e., transcriptomics, proteomics, and genotype–phenotype association studies. Expression-array platforms were installed at AGRF, the Commonwealth Scientific and Industrial Research Organisation (CSIRO), and a number of universities and medical research institutes. Leading statisticians in universities and CSIRO took on the high-dimensional problems posed by expression array data. The word proteome was coined in 1994 by Marc Wilkins, then a PhD student at The University of Sydney [3]. Proteomics developed around mass spectrometry and separation platforms, with activity at Macquarie University in Sydney giving rise in 1995 to the MNRF Australian Proteome Analysis Facility (APAF), and in 1999 to the for-profit company Proteome Systems Ltd [4]. Like AGRF, APAF was developed with several specialist nodes located in different Australian capital cities.

In 2000, Australian bioinformatics (specifically ANGIS, the Australian National Genome Information Service) was seen to be a world leader in bioinformatics service provision [1]. With Internet connectivity to the National Center for Biotechnology Information (NCBI) or European Bioinformatics Institute (EBI) slow and expensive, ANGIS—founded by one of us (TL) at The University of Sydney in 1990—provided an innovative service delivery model based on local data mirrors, user-friendly portals, nodes in major cities, and a national training program. University departments as a whole, as well as individual researchers from academia, the public sector, and private industry, subscribed to the ANGIS service, and it was used intensively for molecular biology and bioinformatics education across Australia. ANGIS was a founding member of Asia-Pacific BioNet, formed in 1998 based on the EMBNet model of distributed data and computational resources embedded with major user communities [1], and itself served as a model for the Canadian Bioinformatics Resource. A BioMirror site was established at the Australian National University (ANU) in coordination with the Australian Partnership in Advanced Computing (APAC) to provide data, but was not intended to offer analysis or querying functionality to biologists.

Beginning in 1998, high-performance computing in Australia was organised through APAC and its eight partners (one in each state of Australia, one in the national capital Canberra, and one in CSIRO). Researchers accessed the APAC national supercomputing facility and most partner facilities via a semiannual peer-reviewed application scheme which continues, with improvements, to the present. APAC also operated support and outreach programs in advanced computing, and from 2004 instituted discipline-based projects, including one in bioinformatics, to build familiarity with grid technologies within communities and to trial common solutions.

This background sets the stage for our description of bioinformatic and computational genome-scale biology in Australia in 2008. While the focus of this paper is science, we must first refer to the policy framework that, perhaps more keenly now than at most other points in recent history, is shaping Australian computational bioscience. Beginning in 2000, a series of high-level discussion papers, government reports, and strategy documents [5]–[12] considered how to strengthen and provide cohesion to Australia's bioinformatics effort. These culminated in a 2005 national bioinformatics strategy [13] that forms part of the national biotechnology strategy. The bioinformatics strategy document made 41 “key findings” and proposed recommendations in infrastructure, research and development, education and training, commercialisation, data management, and coordination. In a parallel initiative, in 2004 two Federal Government ministries jointly constituted the national e-Research Coordinating Committee to make recommendations on how best to facilitate the application of information and communication technologies (ICT) across Australian R&D [14]. Also in 2004, following on from earlier recommendations [5], the Federal Government implemented a top-down strategic infrastructure funding process in fifteen identified capability areas including “evolving bio-molecular platforms and informatics” [15]. Together with co-investment by the States and CSIRO, this National Collaborative Research Infrastructure Strategy (NCRIS) will allocate more than AUD 1 billion from early 2007 through mid 2011. National support for AGRF (genomics), APAF (proteomics), and APAC (advanced computing) is being subsumed under NCRIS, and new capabilities are being funded. In early 2008 the structural changes implemented through NCRIS are beginning to impact Australian research communities, including those involved in bioinformatics and computational genome-scale biology.

## Computational Biology Research in Australia: Centres and Programs

One readily apparent change to the face of Australian research since the Millennium has been the advent of large integrated bioscience institutes and centres with significant computational and/or informatic focus. Some of these are centralised physical centres (e.g., the first three described below); others exist within a geographically distributed organisation, and some are virtual centres involving multiple organisations.

The Bio21 Institute, established in Melbourne in 2000, is a joint initiative of 20 universities, institutes, hospitals, and centres in the state of Victoria founded by Melbourne Health (MH), the University of Melbourne, and the Walter and Eliza Hall Institute of Medical Research (WEHI). Bio21 aims to build synergies in biomedical research and biotechnology based on shared technologies (proteomics, protein structure determination, high-throughput chemical screening, metabolomics, informatics), business development, and training initiatives. Bio21 has a major bioinformatics research component that is being integrated with human clinical data. One initiative is the informatics platform MMIM (Molecular Medicine Informatics Model), which integrates clinical, patient outcome, and genomic data across eight of the partners, including a major healthcare provider (MH), three hospitals, two research institutes (WEHI, and the Ludwig Institute for Cancer Research), and a clinical trials network (Cancer Trials Australia). Six further healthcare sites are expected to join MMIM by the end of 2009 (see Box 2).

Also established in 2000, the Institute for Molecular Bioscience (IMB) of The University of Queensland, in Brisbane, brings together some 500 research staff and postgraduate students in cellular, developmental, structural, and genomic biology of human, mouse, and zebrafish, and in immediately related areas of medicinal chemistry and preclinical drug development. Some 60 of these personnel are engaged directly in computational molecular biology and bioinformatics, the largest such grouping in Australasia. Projects encompass the discovery of regulatory motifs [16] and ncRNAs [17]; computational analysis of membrane proteins and intracellular trafficking [18]; modelling genetic regulatory networks [19] and dynamic spatial processes in endosomes and membranes [20]; comparative genomics [21]; and transcriptome analysis [22] including the use of SOLiD sequencing. IMB also serves as the headquarters of the Australian Research Council (ARC) Centre of Excellence in Bioinformatics, the Queensland Facility for Advanced Bioinformatics, and the bioinformatics component of the Australian Centre for Plant Functional Genomics (ACPFG) (see Box 3).

The Biosciences Research Centre (BRC) at Latrobe University, in Melbourne, is a partnership between the university and the Department of Primary Industries in the state of Victoria (http://hornbill.cspp.latrobe.edu.au/). The aims of BRC are to leverage gene discovery technologies to identify candidates for agricultural crop improvement, and to address strategies for agricultural sustainability in the face of climate change. The current staffing of more than 100 is planned to grow to 450, including further expansion in genomics and bioinformatics. BRC's discovery effort is supported by computational gene annotation and data integration across ESTs, annotated genomes, microarrays, genotyping, phenotype, and plant breeding experiments. This centre is, in turn, a partner within the Victorian Bioinformatics Consortium (http://www.vicbioinformatics.com/home.shtml) based at Monash University.

Other major institutes and centres with a significant computational bioscience profile include Sydney's Garvan Institute of Medical Research (specifically the Peter Wills Bioinformatics Centre: http://linkage.garvan.unsw.edu.au/bioinformatics/), the WEHI (microarray statistics, statistical genetics: http://bioinf.wehi.edu.au/), and the Western Australian Institute of Medical Research (WAIMR) in Perth (http://www.amber.org.au/). Research at WAIMR focuses on genetic epidemiology, and researchers there have access to important cohorts for population genetic data (see Box 4).

Investment in biomedical bioinformatics continues to grow in Australia. For instance, in 2006 the Australian National Health and Medical Research Council (NHMRC) awarded eight large grants under its Medical Bioinformatics Genomics and Proteomics Program in areas of functional genomics, dendritic cell heterogeneity profiling, phosphoproteomics, genome-wide linkage-association scans, retroviral expression cloning, and disease gene mapping in populations (http://www.nhmrc.gov.au/FUNDING/funded/outcomes/medbio.htm).

The CSIRO, Australia's national science agency, conducts research in bioinformatics and computational bioscience and applies bioinformatics technologies in at least eight divisions and centres (Entomology, Food Science, Information and Communications Technology, Livestock Industries, Marine and Atmospheric Research, Mathematical and Information Sciences, Molecular and Health Technologies, and Plant Industry) around Australia (see Box 5). A broad range of partner and internally generated projects include genome sequencing and annotation, functional and epigenomics, rumen and environmental metagenomics, marker-assisted breeding and trait association, genomic approaches to disease resistance, desiccation and salinity tolerance, environmental and climate modelling, and characterisation of biodiversity.

National ICT Australia (NICTA) was established in 2002 to conduct and coordinate aspects of Australia's research in information and communications technologies. With more than AUD 250 million in federal government funding plus significant co-investment, NICTA researchers carry out and collaborate on research in networks, embedded systems, data mining, and complexity. Bioscience and medicine represent significant application domains, with NICTA involvement in automated feature extraction from MRI scans, body area sensor networks, expression analysis in cancer detection and treatment monitoring, large-scale marker-assisted breeding of crop plants, and network visualisation (http://www.cs.usyd.edu.au/visual/valacon/).

The ARC Centre of Excellence in Bioinformatics (http://bioinformatics.org.au/) supports coordinated research programs in five Australian universities: The University of Queensland (eight research groups), The University of Newcastle (two), Deakin University, Macquarie University, and ANU (one each). The Centre's research focuses in four areas: (1) the Visible Cell project in conjunction with IMB; (2) inference of pathways and regulatory networks based on molecular interaction motifs, gene expression data and ncRNAs; (3) computational modelling of dynamic processes in eukaryotic cells and biomembranes, again in conjunction with IMB; (4) the development and application of FPT [23] and metaheuristic algorithms for very large problems on graphs and networks. The Centre also includes three overseas partner institutions (IBM TJ Watson Research Center, the University of Auckland, and the University of Tennessee) and has other active international research collaborations including a European supercomputing grid project (http://www.qoscosgrid.eu/), and a semantic modelling project with Pfizer Research Technology Center in Cambridge, Massachusetts (http://www.biomanta.org/).

The Visible Cell project (http://www.visiblecell.com/) within the ARC Centre of Excellence in Bioinformatics aims to inform in silico studies of cell and molecular organisation in three dimensions, using the mammalian cell as a unitary example of an ordered complex system. This unique initiative is based on whole-cell tomography of serially sectioned cells—at this point the insulin-producing beta cell—imaged at medium (15–20 nm) and high (4–5 nm) resolution using electron tomography [24]. Gene products, molecular interactions, pathways, networks, and processes are localised to the appropriate cellular coordinates via an ontologically aware semantic querying interface and federated data supplied by the Queensland Facility for Advanced Bioinformatics (see Box 6). The goal is to “change the way we think about mammalian cells” by allowing biologists to visualise genome-scale data, and to model and simulate molecular processes, not in abstract spatial coordinates but within reconstructions of actual cells.

At least two other ARC-funded research centres have significant computational biology or informatic components. The ARC Centre of Excellence for Kangaroo Genomics (ANU, University of Melbourne, University of New South Wales, WEHI, and AGRF) focuses on comparative genomics and genomic biology of the tammar wallaby *Macropus eugenii*. Project areas include large-scale annotation of marsupial genomes, curated annotation of targeted genomic regions (e.g., MHC and XIST loci), and the development and application of comparative methods for the analysis of genomic regions that do not encode proteins. The ARC Centre of Excellence in Structural and Functional Microbial Genomics (http://www.microbialgenomics.net/), headquartered at Monash University, applies expression technology, proteomics, protein crystallography, and bioinformatics toward the understanding of host–pathogen interactions and the development of vaccines and drugs for selected microbial pathogens including *Burkholderia pseudomallei*, *Clostridium perfringens*, *Escherichia coli*, and species of *Legionella*, *Leptospira*, and *Mycobacterium*.

Several research-intensive universities have established centres of computational molecular or genomic science. The Centre for Bioinformation Science, established in 2000 and based in the Mathematical Sciences Institute at ANU, focuses on mathematical and statistical aspects of molecular bioscience including expression array analysis and molecular evolution (http://dayhoff.anu.edu.au/). The Western Australia Centre for Comparative Genomics (CCG) at Murdoch University was established in 2006, in part as a continuation of the Centre for Bioinformatics and Biological Computing. CCG pursues research in human, plants, animals, pathogens, and bioinformatics, and hosts the Australian Bioinformatics Facility established under NCRIS to provide bioinformatics support for national omics platforms (see Box 7). The Newcastle Priority Research Centre for Bioinformatics, Biomarker Discovery, and Information-Based Medicine, based at the Hunter Medical Research Institute and the nearby University of Newcastle, was founded in 2006 to develop novel analytical strategies, particularly based on combinatorial optimisation and metaheuristic algorithms, and to apply high-throughput genomic and proteomic technologies toward the improvement of clinical outcomes through individualised treatment (http://livesite.newcastle.edu.au/cibm). Sydney Bioinformatics, formed in 2007 through the merger of the Sydney University Biological Informatics and Technology Centre and ANGIS, carries out research in theoretical and applied bioinformatics and continues the bioinformatics infrastructure and advanced training activities of ANGIS (http://www.usyd.edu.au/sydneybioinformatics/). At the University of New South Wales, also in Sydney, a Quantitative and Computational Biology research grouping focuses mathematical and modelling techniques on problems in gene regulation and expression, proteomics, immunology, population genetics, epidemiology, and viral evolution (http://www.babs.unsw.edu.au/research/qcb/index_qcb.html).

##

**Box 2. Molecular Medicine Informatics Model**

One of the key challenges in realising the human health benefits of genomics and bioinformatics is to access large amounts of clinical data from well-described patient cohorts. For genotype association studies to achieve statistical power, and to reduce the impact of noise created by genomic variation, large numbers of patients are needed. If thousands or tens of thousands of patients are required, this cannot be done within the boundaries of a single institution. This philosophy forms the foundation of the Molecular Medicine Informatics Model (MMIM) project, which has established a technological and policy framework to enable hospitals to share de-identified patient data for research purposes (http://www.mmim.org.au/pages/index.php). MMIM is a collaborative venture between the Bio21 Institute and Melbourne Health in the state of Victoria. The technology federates existing data sources across hospitals throughout the state and from across the country into a single interface. The data are updated daily, and an end-user can instantly join data from across institutions and databases for cancer, diabetes, epilepsy, and stroke. The MMIM policy framework provides a common mechanism to satisfy ethics, privacy, security, and intellectual property. The project thus provides a data platform for researchers to access and share clinical and genetic data on patients across institutions to drive advances in our understanding of human disease processes. —*Bruce Ross*

**Box 3. Computational Biology in ACPFG**

The Australian Centre for Plant Functional Genomics (http://www.acpfg.com.au/) was established in 2003 to provide national capability in cereal genomics. ACPFG is intended to discover genes and gene systems, develop technologies and access others from overseas, and deliver to the Australian farming community cereal varieties with improved tolerance to abiotic stress. ACPFG is headquartered at The University of Adelaide's Waite Campus and has research nodes at The University of Melbourne and The University of Queensland. In total, ACPFG employs more than 110 staff.

The University of Queensland node is dedicated to bioinformatics development supporting ACPFG research; the presence of additional support groups at each of the other nodes ensures tight integration of bioinformatics within research programs. These bioinformatics groups manage data produced within ACPFG, provide access to external data, and work with laboratory researchers to develop novel tools and interfaces for robust analysis.

Plants produce a vast range of primary and secondary metabolites, many of which have agronomic or medical applications. High-throughput characterisation of these compounds is being undertaken at the Melbourne node using advanced mass spectrometry. In support, the Melbourne node is developing custom databases and tools to store and analyse metabolomic data, and to integrate the results with data from gene-expression studies at the Adelaide node. Crop genomes are generally very large, frequently polyploid, and characterised by abundant repetitive elements, making genome sequencing a major challenge. Bread wheat, for example, has a hexaploid genome more than five times the size of the human genome, with the excess due mostly to the expansion of families of repetitive elements. Research at the Brisbane node has focussed on development of methods for assembling DNA sequence from Applied Biosystem's next-generation SOLiD system, to enable the cost-effective sequencing of large, complex genomes. Automated sequence annotation pipelines include tools for the identification of molecular genetic markers that are being applied within ACPFG to associate genes with important agronomic traits and for marker-assisted breeding of crop varieties that maintain productivity in an often unpredictable and harsh environment.

The ACPFG bioinformatics groups work closely with plant phenomic and metabolomic facilities under NCRIS, the ARC Centre of Excellence in Bioinformatics, and the Queensland Facility for Advanced Bioinformatics. —*Dave Edwards, Head of Bioinformatics, ACPFG*

**Box 4. Joondalup**

Human disease is a product of the combination of genetic makeup and environmental triggers. Most genetic association studies focus on the correlation of genetics with the disease phenotype after disease manifestation, and take little account of the factors that predispose disease occurrence or environmental associations. Longitudinal studies of populations give a long-term, multifactorial view of humans and disease. In order to address this issue, the Western Australian Institute for Medical Research (WAIMR) has started the Joondalup Family Health Study that aims to follow 80,000 citizens every three years in the city of Joondalup (http://www.jfhs.org.au/). The data collected will comprise comprehensive clinical and questionnaire data, with an aim to collect 3,000 data points for each three-year time point. The focus is on families to enable pedigree analytical techniques to be used as well as population-based genetic association. The project commenced in late 2007 with the aim of collecting data from 2,000 subjects in the first instance. —*Bruce Ross*

**Box 5. 'Omics and Bioinformatics in CSIRO**

The Commonwealth Scientific and Industrial Research Organisation is Australia's national science agency and one of the largest and most diverse research agencies in the world, with more than 50 sites and 6,500 employees throughout Australia and overseas. CSIRO has significant bioinformatics activities based in Mathematical and Information Sciences (CMIS), Livestock Industries (CLI), Plant Industries (PI), Entomology (CEnto), Ensis (a joint venture with Scion, in sustainable forest industries), Food Science Australia (FSA) and the Population Health and Food Futures Flagships. These groups have access to a dedicated computing array and other shared computational infrastructure. The main areas of interest include genome sequencing and assembly, miRNomics, gene expression analysis, whole-genome association analysis from SNPs, and integrated systems biology analyses. Across CSIRO, groups are involved in genome projects on cow and sheep (CLI); grape (PI); eucalypt (Ensis); insects, including honeybee and cotton boll worm (CEnto); large ecogenomics and metagenomics projects (P-Health, CEnto, CLI); and individual fungi (PI) and bacteria (CLI, FSA). A large initiative in epigenetic regulation has been funded as part of a cross-CSIRO Transformational Biology initiative. As part of an Emerging Sciences project, researchers in PI and CLI are studying the miRNome of a number of plants and animals. CMIS has developed a novel vertebrate microarray to facilitate expression analysis for organisms for which the genome has not yet been sequenced.

With the emergence of large SNP-chips and gene association studies combining genotyping and phenotyping, substantial work is under way on colorectal cancer in humans (P-Health) and a range of production and quality phenotypes in cattle (CLI). Large gene expression analyses and methodology development for datasets containing more variables than observations (CMIS) are morphing into systems biology projects with significant projects in colorectal cancer (P-Health), muscle biology (Food Futures), and inference and visualisation of complex networks (CMIS). Complementary research is under way in CMIS on the analysis and extraction of data from complex images of biological samples. —*Brian Dalrymple, Systems and Computational Biology, CLI*

**Box 6. Queensland Facility for Advanced Bioinformatics**

QFAB (http://www.qfab.org/) is a joint initiative of seven institutions and the Government of Queensland to provide advanced capability and facilities in data management, integration, and analysis for Australia's growing biotechnology and health sectors. Founded in early 2007, QFAB is providing high-end data integration and analysis for its partners and a growing base of clients. Its partners are The University of Queensland (UQ), Queensland University of Technology (QUT), Griffith University, eHealth Research Centre (a joint venture of CSIRO and Queensland Government), Queensland Department of Primary Industries and Fisheries (QDPI&F), Queensland Cyber Infrastructure Facility, and the Australian Partnership in Advanced Computing (the latter now incorporated into NCRIS). Partner contributions often leverage other initiatives, including the ARC Centre of Excellence in Bioinformatics at UQ, the Microsoft QUT eResearch Centre, and the QDPI&F Agricultural Biotechnology Centre.

QFAB's partners provide tools, workflow technology, health data integration software, computational resources, and ICT experience. QFAB operates mirrors of the UCSC Genome Browser and ENSEMBL, and hosts public and proprietary datasets federated via SRS. With transparent access to data and applications via Storage Resource Broker, project-dedicated computation and storage, and the security of shibboleth, certificates and keys, partners and clients carry out secure in silico discovery both onsite and remotely.

QFAB has delivered an initial solution to the challenge faced by the KOALA Childhood Obesity Project (http://koala.imb.uq.edu.au/) in capturing data from participating families and researchers, and continues to work with the project toward its goal of identifying biomarkers for the future onset of obesity-related illnesses. QFAB is providing the informatics for a large five-year project on nuclear receptors in breast cancer, led by the Westmead Research Institute in Sydney and supported by the National Breast Cancer Foundation. For key data-integration and collaboration components of the latter project, QFAB is working in partnership with a national e-research project (http://www.archer.edu.au/) recently subsumed under NCRIS. —*Jeremy Barker, CEO QFAB*

**Box 7. Australian Bioinformatics Facility**

The Australian Bioinformatics Facility (ABF) supports the Australian Government's AUD 50 million investment in “omics” and bioinformatics through the National Collaborative Research Infrastructure Scheme (NCRIS). ABF delivers leading-edge bioinformatics infrastructure and services to build bioinformatics service capacity within and across the three national omics platforms (genomics, proteomics, and metabolomics). ABF supports these platforms in three main ways:

- Working in collaboration with the platforms, ABF will embed bioinformaticians in the platform nodes at locations across Australia. These embedded bioinformaticians will support existing bioinformatics staff in the nodes and will work across a myriad of platform-specific projects.
- From its Perth base, ABF will provide direct (non-embedded) bioinformatics support to platform staff across a range of projects agreed between the platforms and ABF. These non-embedded staff will facilitate cross-platform initiatives to minimise duplication and maximise output associated with the bioinformatics investment.
- Through linkages with bioinformatics facilities nationally and internationally, ABF will disseminate best practice to platforms to enhance their bioinformatics service capacity.

ABF is a specialised service platform operating within CCG and drawing on its full range of expertise and infrastructure. CCG (http://ccg.murdoch.edu.au/index.php/Main_Page) represents a unique approach to research in comparative genomics, drawing together biomedical and agricultural research and development, bioinformatics activities, and expertise in comparative genomics to promote shared understanding within and across fields of study.

The CCG team has expertise in bioinformatics, computational biology, comparative genomics, molecular biology, Internet-based software development, and high-performance computing. Specific bioinformatics capabilities include sequence assembly, gene and genome annotation, mapping, transcriptomics, comparative genomics, vaccine candidate identification, new tools for alignment, phylogenetic analysis, gene expression and statistical analysis, peptide mass fingerprinting analysis, and customisation of protein datasets. At the heart of CCG is the Bioinformatics Research Laboratory (BRL), which supports academic and industry researchers with a range of capabilities including the development of computational tools, data analysis and high-resolution visualisation strategies, and integrated Web-based applications. BRL is a dedicated high-performance computing facility that will soon be upgraded to 500+ cores. A key feature is a 4×3 m, 18 million pixel high-resolution display wall. BRL also provides industry and scientists access to state-of-the-art computing infrastructure and support. —*Matthew Bellgard, Convenor of the Australian Bioinformatics Facility, and Director of CCG*

## Participation in International Programs

Australian researchers and research organisations play proactive roles in a number of international genomics projects, including the FANTOM3 and FANTOM4 mouse transcriptome projects with RIKEN Genomic Sciences Centre [25],[26], bovine, sheep, insect, and other genome projects (see Box 5), marsupial genomics including the tammar wallaby project (ARC Centre of Excellence for Kangaroo Genomics), sponge genomics [27], the *Plasmodium* [28] and *Trichomonas* genomes [29], and wastewater metagenomics [30]. Projects based mostly within Australia include scabies cDNA sequencing [31] and developing work on gut metagenomics.

In late 2007, Australia became an associate member of the European Molecular Biology Laboratory (EMBL). Dr Nadia Rosenthal, Director of the EMBL outstation in Monterotondo, near Rome (Italy), is relocating part of her research to Monash University, in Melbourne, where she will direct the new Australian Regenerative Medicine Institute. Other new initiatives, including Australia-funded group leaders in Europe and EMBL laboratories in Australia, will be coordinated through the new body EMBL Australia. The founding members of EMBL Australia are CSIRO, four research-intensive universities (Monash, Queensland, Sydney, and Western Australia), and the federal government through NCRIS.

Modelled after EMBL, the electronic International Molecular Biology Laboratory (eIMBL) is a Korean initiative with virtual research groupings or study groups in six areas (http://www.eimbl.org/). The most active of these, in Systems Biology, links systems researchers in ten countries, including one group in Australia (at IMB). eIMBL emphasises the training of young researchers, exchange of knowledge and resources, and collaboration in systems biology research using Web/grid infrastructure.

Australia has hosted important international conferences in computational biology and bioinformatics including the inaugural Asia-Pacific Bioinformatics Conference (APBC-2003) in Adelaide, the 11^th^ International Conference on Intelligent Systems in Molecular Biology (ISMB-2003) in Brisbane, and the inaugural Computational Models for Life Sciences (CMLS'07) on the Gold Coast. The 19^th^ International Conference on Genome Informatics (GIW-2008) will be held on the Gold Coast in early December 2008. Computational biology is increasingly finding a place in other international events in Australia, including conferences in genomics, high-performance and grid computing, and e-research.

## Infrastructure, Education, Training, and Career Development

As described above, the 2005 national bioinformatics strategy [13] made recommendations in several areas including infrastructure. During 2007, Bioinformatics Australia (BA), the national peak body in bioinformatics (see Coordination, below), conducted extensive face-to-face consultation in five cities to assess infrastructure needs in detail. These needs fell into three areas: (1) hard infrastructure (computing hardware and networks); (2) soft infrastructure (software tools, data mirrors, data-exchange formats); (3) human infrastructure (people skilled in various bioinformatics areas), with the latter identified as the most pressing issue. To the extent that hard or soft infrastructure was underutilised or did not fully address the needs of the biosciences R&D community, this was invariably seen to be due to limitations on human expertise, not on the hard or soft infrastructure components per se. These human infrastructure needs can be addressed via education and training. Three levels of education and training need were identified: (1) undergraduate education in a core area such as molecular bioscience, computer science, or information technology, with supplementary work in the cognate disciplines; (2) postgraduate education including coursework as well as research; (3) short courses to up-skill researchers from both life sciences and informatics backgrounds.

As many as 14 Australian universities offer undergraduate degree programs or streams in computational biology and/or bioinformatics ([32], updates on http://www.ausbiotech.org/bioinformatics, http://www.nslij-genetics.org/bioinfotraining/, and elsewhere). A level of dynamism is expected in any competitive field, but Australian tertiary education programs are currently unusually dynamic. The University of Melbourne (http://www.futurestudents.unimelb.edu.au/about/m_model/index.html) is replacing its nearly 100 existing undergraduate degrees with a US-style three-year Bachelors in one of six broad areas (e.g., Science) followed by specialised Masters or PhD training. Several other research-intensive universities are either considering this model, or consolidating degree programs without adopting the model fully. As a consequence, courses in computational molecular biology and bioinformatics could be developed at the postgraduate, not undergraduate, level. Already the market demand is greater for graduates with PhD and MSc degrees in these areas than for undergraduate degree-holders. Market demand extends across software development, data management, statistics, and life sciences and health application domains. Despite the market demand for skilled bioinformaticians and the availability of diverse educational programs (mostly at the undergraduate level), uptake by students is currently low. The lack of clear career paths in bioinformatics was identified as the most likely reason for this mismatch between supply and demand.

The BA survey found a substantial degree of satisfaction with the (mostly) generic personnel support that has been provided by national hard-infrastructure facilities such as APAC. As data management and interpretation challenges increase, however, there may be scope for more-specialised facilities such as Queensland Facility for Advanced Bioinformatics (QFAB) (Box 6) that provide a customised, integrated approach to hard infrastructure, software, and human expertise. BA also heard concerns that many integrated bioinformatics software packages require specialised skills; facilities such as ANGIS remain popular because they provide and maintain user-friendly environments such as the Web-based BioManager bioinformatics system.

Professional bioinformatics training remains in demand in Australia. The Ludwig Institute for Cancer Research, in Melbourne, has run a bioinformatics training course since 1998. A week-long “BioInfoSummer” workshop, held annually since 2003 at ANU in Canberra, presents topics in bioinformatics to students and researchers from diverse backgrounds. The one-week Winter School in Mathematical and Computational Biology, organised in late June or early July since 2004 by the ARC Centre of Excellence in Bioinformatics, covers areas beyond bioinformatics per se, including algorithmics, computational neuroscience, and e-research. Each of the latter two advanced schools typically attracts 150–200 participants. ANGIS has offered training courses in the use of bioinformatics tools across Australia, throughout the year, since its inception in 1990.

The spread of high-throughput “omic” technologies, including next-generation DNA sequencing, various array platforms, metabolomics, and mutagenesis screens in zebrafish or mouse is already placing new demands on human infrastructure, some but not all of which are being addressed within NCRIS (above). In part through NCRIS, the Australian government is taking a coordinated national approach to management of the research data lifecycle, including capture, interoperation, access, and support for collaboration, with explicit recognition of the key role of expert human infrastructure.

## Commercial Activity

Australia is home to some 427 biotechnology companies [33] of which 49% are involved in human therapeutics, 16% in agricultural biotech, and 13% in diagnostics; these numbers exclude the 625 companies that focus on medical devices. Several of these companies have significant positions in computational biology, computational chemistry, bioinformatics, or statistical genetics. Catapult Genetics (http://www.catapultgenetics.com/), recently formed by the merger of the Brisbane-based Genetic Solutions with New Zealand-based Catapult Systems, develops DNA markers and other molecular and informatic technologies for product identification, validation, traceability, quality enhancement, and production efficiency in cattle and sheep. ChemGenex Pharmaceuticals (http://www.chemgenex.com), based in Geelong (near Melbourne), uses genotyping of disease pedigree cohorts to identify genomic regions associated with diabetes and obesity; after fine-scale mapping and gene identification, validation is carried out in animal models. Nucleics (http://www.nucleics.com/), based in Bendigo (also near Melbourne), conducts research on new technologies for DNA sequencing and epigenomics, including software and bioinformatics. Sydney-based Proteome Systems (http://www.proteomesystems.com/) develops protein biomarkers associated with diseases with focus on tuberculosis, prostate cancer, and Huntington Disease, and following the acquisition of a US company have a portfolio of small molecule drug candidates, some of which are in clinical trials. The well-regarded BioinformatiQ software suite for proteomics was developed by Proteome Systems. In addition, many global pharmaceutical firms, such as Johnson & Johnson Research (http://www.johnsonandjohnson.com.au), have a strong computational or informatic R&D presence in Australia.

The business case in bioinformatics per se is (as elsewhere internationally) more challenging. Early companies included eBioinformatics, a startup spun out from The University of Sydney, and BioLateral, followed later by CSIRO Bioinformatics, a wholly owned subsidiary of CSIRO, and the privately held Emphron Informatics (http://www.emphron.com) in Brisbane.

## Coordination

The national peak bioinformatics body in Australia is BA. BA was formed in 2004 by the merger of the Association of Australian Bioinformatics (AAB), an academic-based society, with the Bioinformatics Special Interest Group within AusBiotech (the national biotechnology peak body). BA is legally a subentity of AusBiotech (http://www.ausbiotech.org/) but operates in many regards as an independent entity; decisions for BA are made by a Management Committee of seven members elected by the BA membership plus two appointed by AusBiotech. Decisions involving a direct and specific financial or contractual commitment must be ratified by AusBiotech before they can be actioned. Two of the authors (TL and MAR) are BA president and vice-president, respectively. Each of BA's first two annual conferences (Sydney 2006 and Brisbane 2007) attracted 100–110 participants. The 2008 BA conference will be combined with the International Conference on Genome Informatics (GIW-2008), which will be held on the Gold Coast from 1–3 December 2008.

One of the outcomes of the 2005 bioinformatics strategy [13] was federal funding for an Australian Bioinformatics Network (2006–2007) managed by BA. This allowed BA to appoint a coordinator, conduct a nationwide survey of needs and concerns among the bioinformatics R&D community (see Infrastructure, Education, Training, and Career Development, above), and establish a Web site (http://www.ausbiotech.org/content.asppageid77).

BA (in its earlier incarnation as AAB) was a founding partner within the Association of Asian Societies of Bioinformatics (AASBi), a voluntary partnership among bioinformatics societies in Japan, South Korea, Singapore, Taiwan, and Australia. AASBi has taken responsibility for the annual International Genome Informatics Conference (GIW) and will develop other initiatives in the region.

## Envoi

Set against the previous review in 2000 [1], the landscape of computational biology and bioinformatics in Australia has evolved almost (but not quite) beyond recognition. Genome-scale R&D has arrived, although program continuity remains an issue, and the Australian community lacks a common institutional base comparable to EBI or NCBI. Nonetheless, much high-quality research, both fundamental and applied, is under way, and bioinformatics has been recognised [34] as an area of emerging strength critical to the country's established life sciences and medical sectors. Recommendations in the national bioinformatics strategy have been refined via the Australian Bioinformatics Network managed by the new national peak body, Bioinformatics Australia. Institutions across the country offer a range of undergraduate and postgraduate educational opportunities. Hard and soft infrastructure is adequate, but ongoing commitment will be required to support next-generation sequencing and systems biology. More remains to be done in advanced training, the development of human expertise, and the clarification of career paths. Through NCRIS and associated initiatives, Australia is showing leadership in the development and implementation of coordinated national systems for integrated access, high-performance computing, and end-to-end management of the data lifecycle. Many international links are in place (with more under development), and the commercial sector—which has much to benefit from growth and consolidation of computational biology and bioinformatics—is supportive. Community-building is under way on numerous fronts. Although much remains to be done, genome-scale computational biology and bioinformatics in Australia is on a very promising trajectory.

## References

[1] Littlejohn T (2000). Bioinformatics in Australia.. Bioinformatics.

[2] Bulach DM, Zuerner RL, Wilson P, Seemann T, McGrath A (2006). Genome reduction in *Leptospira borgpetersenii* reflects limited transmission potential.. Proc Natl Acad Sci U S A.

[3] Wasinger VC, Cordwell SJ, Cerpa-Poljak A, Yan JX, Gooley AA (1995). Progress with gene-product mapping of the Mollicutes: *Mycoplasma genitalium*.. Electrophoresis.

[4] Mathews JA, del Carmen R (2002). Proteome Systems Ltd: A Macquarie life-sciences spinoff. Macquarie Graduate School of Management (MGSM) Case 2002-2.. http://www.mgsm.edu.au/download.cfmDownloadFileC0228623-FFA9-4047-BC8F2C8339D17E36.

[5] Batterham R (2000). The chance to change—A discussion paper. Office of the Chief Scientist Australia.. http://bioinformatics.org.au.

[6] National Health and Medical Research Council Research Committee, Australian Research Council, and Commonwealth Scientific and Industrial Research Organisation (2000).

[7] House of Representatives Standing Committee on Primary Industries and Regional Services (2001). Bioprospecting: Discoveries changing the future (“the Bailey Inquiry”). Canberra.. http://bioinformatics.org.au.

[8] Department of Industry, Tourism, and Resources (2002). Bioinformatics: Issues and opportunities for Australia. Emerging Industries Occasional Paper No. 15 “the Littlejohn Report”. Canberra: ITR.. http://bioinformatics.org.au.

[9] National Health and Medical Research Council Research Committee, Australian Research Council, Commonwealth Scientific and Industrial Research Organisation, and Biotechnology Australia (2002).

[10] National Health and Medical Research Council Research Committee, Australian Research Council, Commonwealth Scientific and Industrial Research Organisation, Biotechnology Australia, Environment Australia (2002).

[11] Department of Industry, Tourism, and Resources (2003).

[12] Department of Industry, Tourism, and Resources (2004).

[13] Biotechnology Australia (2005). National bioinformatics strategy. Canberra.. http://bioinformatics.org.au.

[14] Department of Education, Science, and Training, and Department of Communications, Information Technology, and the Arts. e-Research Coordinating Committee (2005). An Australian e-research strategy and implementation framework. Final report of the e-Research Coordinating Committee. Canberra.. http://bioinformatics.org.au.

[15] National Collaborative Research Infrastructure Strategy (NCRIS) Committee (2006). National Collaborative Research Infrastructure Strategy. Strategic Roadmap. Canberra.. http://bioinformatics.org.au.

[16] Redhead E, Bailey TL (2007). Discriminative motif discovery in DNA and protein sequences using the DEME algorithm.. BMC Bioinformatics.

[17] Mehler MF, Mattick JS (2007). Noncoding RNAs and RNA editing in brain development, functional diversification, and neurological disease.. Physiol Rev.

[18] Sprenger J, Fink JL, Karunaratne S, Hanson K, Hamilton NA (2008). LOCATE: A mammalian protein subcellular localization database.. Nucleic Acids Res.

[19] Tian T, Burrage K (2006). Stochastic models for regulatory networks of the genetic toggle switch.. Proc Natl Acad Sci U S A.

[20] Burrage K, Hancock J, Leier A, Nicolau DV (2007). Modelling and simulation techniques for membrane biology.. Brief Bioinform.

[21] Beiko RG, Harlow TJ, Ragan MA (2005). Highways of gene sharing in prokaryotes.. Proc Natl Acad Sci U S A.

[22] Forrest AR, Taylor DF, Crowe ML, Chalk AM, Waddell NJ (2006). Genome-wide review of transcriptional complexity in mouse protein kinases and phosphatises.. Genome Biol.

[23] Downey TG, Fellows MR, Langston MA (2008). The Computer Journal special issue on parameterized complexity: Foreword by the guest editors.. Comp J.

[24] Marsh BJ (2007). Reconstructing mammalian membrane architecture by large area cellular tomography.. Meth Cell Biol.

[25] Carninci P, Kasukawa T, Katayama S, Gough J, Frith MC (2005). The transcriptional landscape of the mammalian genome.. Science.

[26] Carninci P, Sandelin A, Lenhard B, Katayama S, Shimokawa K (2006). Genome-wide analysis of mammalian promoter architecture and evolution.. Nat Genet.

[27] Jackson DJ, Macis L, Reitner J, Degnan BM, Wörheide G (2007). Sponge paleogenomics reveals an ancient role for carbonic anhydrase in skeletogenesis.. Science.

[28] Bahl A, Brunk B, Coppel RL, Crabtree J, Diskin SJ (2002). PlasmoDB: The *Plasmodium* genome resource. An integrated database providing tools for accessing, analysing, and mapping expression and sequence data (both finished and unfinished).. Nucleic Acids Res.

[29] Carlton JM, Hirt RP, Silva JC, Wang CC, Dunne RL (2007). The draft genome sequence of the sexually transmitted pathogen *Trichomonas vaginalis*.. Science.

[30] Martin HG, Ivanova N, Kunin V, Warnecke F, Barry KW (2006). Metagenomic analysis of two enhanced biological phosphorus removal (EBPR) sludge communities.. Nat Biotech.

[31] Fischer K, Holt DC, Harumal P, Currie BJ, Walton SF (2003). Generation and characterization of cDNA clones from *Sarcoptes scabiei var. hominis* for an expressed sequence tag library; identification of homologues of house dust mite allergens.. Am J Trop Med Hyg.

[32] Cattley S (2004). A review of bioinformatics degrees in Australia.. Brief Bioinform.

[33] Hopper K, Thorburn LJ (2007). BioIndustry Review 2007—Australia and New Zealand.

[34] Prime Minister's Science Engineering and Innovation Council (2006). Australia's Science and Technology Priorities for Global Engagement. Canberra: PMSEIC.. http://bioinformatics.org.au.

